# Maternal Vitamin D Status and Infant Infection

**DOI:** 10.3390/nu10020111

**Published:** 2018-01-23

**Authors:** Sara Moukarzel, Marlies Ozias, Elizabeth Kerling, Danielle Christifano, Jo Wick, John Colombo, Susan Carlson

**Affiliations:** 1 Department of Pediatrics, University of California San Diego, 9500 Gilman Dr, La Jolla, CA 92093, USA; 2 Larsson-Rosenquist Foundation Mother-Milk-Infant Center of Research Excellence, Health Sciences, University of California San Diego, 9500 Gilman Dr, La Jolla, CA 92093, USA; 3 Immunoscience Medical Affairs, Bristol-Myers Squibb, 345 Park Avenue, New York, NY 10154, USA; mkozias@gmail.com; 4 Department of Dietetics and Nutrition, University of Kansas Medical Center, 3901 Rainbow Blvd, Kansas City, KS 66160, USA; ekerling@kumc.edu (E.K.); Scarlson@kumc.edu (S.C.); 5 Department of Neurology, University of Kansas Medical Center, 3901 Rainbow Blvd, Kansas City, KS 66160, USA; dchristifano@kumc.edu; 6 Department of Biostatistics, University of Kansas Medical Center, 3901 Rainbow Blvd, Kansas City, KS 66160, USA; jwick@kumc.edu; 7 Department of Psychology, University of Kansas Medical Center, 3901 Rainbow Blvd, Kansas City, KS 66160, USA; colombo@ku.edu

**Keywords:** infection, 25-hydroxycholecalciferol, vitamin D, pregnancy, infancy, maternal nutrition, African Americans

## Abstract

Maternal vitamin D status during pregnancy may modulate fetal immune system development and infant susceptibility to infections. Vitamin D deficiency is common during pregnancy, particularly among African American (AA) women. Our objective was to compare maternal vitamin D status (plasma 25(OH)D concentration) during pregnancy and first-year infections in the offspring of African American (AA) and non-AA women. We used medical records to record frequency and type of infections during the first year of life of 220 term infants (69 AA, 151 non-AA) whose mothers participated in the Kansas University DHA Outcomes Study. AA and non-AA groups were compared for maternal 25(OH)D by Mann–Whitney *U*-test. Compared to non-AA women, AA women were more likely to be vitamin D deficient (<50 nmol/L; 84 vs. 37%, *p* < 0.001), and more of their infants had at least one infection in the first 6 months (78.3% and 59.6% of infants, respectively, *p* = 0.022). We next explored the relationship between maternal plasma 25(OH)D concentration and infant infections using Spearman correlations. Maternal 25(OH)D concentration was inversely correlated with the number of all infections (*p* = 0.033), eye, ear, nose, and throat (EENT) infections (*p* = 0.043), and skin infection (*p* = 0.021) in the first 6 months. A model that included maternal education, income, and 25(OH)D identified maternal education as the only significant predictor of infection risk in the first 6 months (*p* = 0.045); however, maternal education, income, and 25(OH)D were all significantly lower in AA women compared to non-AA women . The high degree of correlation between these variables does not allow determination of which factor is driving the risk of infection; however, the one that is most easily remediated is vitamin D status. It would be of value to learn if vitamin D supplementation in this at-risk group could ameliorate at least part of the increased infection risk.

## 1. Introduction

Maternal vitamin D status during pregnancy may modulate fetal immune system development, which in turn may alter susceptibility to immune-mediated diseases and infections during infancy [1,2,3,4]. Plasma 25-hydroxycholecalciferol (25(OH)D) is the most widely-accepted biomarker for vitamin D status in humans, although the active form of vitamin D is 1,25-di(OH)D [5]. Several cohort studies to date have implicated low plasma 25(OH)D concentration during pregnancy or at birth with increased risk of respiratory infections in infancy or early childhood [6,7,8,9,10,11,12]. In most cases, diagnoses were made by parent report rather than by a medical practitioner. In addition, none of these studies were conducted in the US, and none included a large number of African-American (AA) participants, who are more likely to be vitamin D deficient compared to women of other races, at least in part because increased skin pigmentation lowers the amount of vitamin D synthesis that occurs with ultraviolet light exposure [5,13,14,15,16,17]. Several studies conducted in countries other than the US have included women who had darker skin or were not Caucasian, but these studies did not specifically look at infection in children of women with darker skin.

We used a cohort of mother–infant dyads to ask if women who were AA differed from those who were not in terms of 25(OH)D concentration and infant infections. We also explored the simple relationship between maternal plasma 25(OH)D and infant infections, in addition to considering socioeconomic variables that could influence risk of infant infection.

## 2. Materials and Methods

### 2.1. Study Participants

This study involved 220 mother–infant dyads who participated in the Kansas University DHA Outcomes Study (KUDOS)—a randomized, double-blind, placebo-controlled clinical trial for which the primary goal was to determine whether prenatal docosahexaenoic acid (DHA) supplementation could improve pregnancy outcomes and infant cognitive development [18]. All pregnancies met the inclusion and exclusion criteria for the KUDOS study; i.e., women were 16–35 years old, had a body mass index (BMI) less than 40, were carrying a single fetus, and were between 8 and 20 weeks (mean 14.4 weeks) gestation when enrolled into the study, and were randomly assigned to placebo or DHA. Any serious illness, high blood pressure, or diabetes at enrollment were exclusion criteria. 

Infants were included in this study if they were generally healthy, born at term (≥37 weeks of gestation), had a complete first-year medical record, and their mothers had a plasma 25(OH)D concentration measured in pregnancy. Out of the 220 infants, 69 infants were born to mothers who were AA and 151 were born to non-AA mothers (135 Caucasian, 12 Hispanic, and 3 Asian).

All aspects of this research were approved by the Institutional Review Boards/Human Subjects Committees at the University of Kansas Medical Center (KUMC; Kansas City, KS, USA); the University of Missouri-Kansas City (Truman Medical Center, Kansas City, MO, USA); and Saint Luke’s Hospital (Kansas City, MO, USA). KUDOS was run under KUMC IRB# HSC 10186 (Birth to 18 months) and HSC 11406 (2–6 years). Data from medical records were authorized for the KUDOS trial and approved for release by infants’ legal caregivers. All participants provided written informed consent at enrollment. 

### 2.2. Data Collection and Biochemical Assessment

Maternal gestational age at enrollment was determined from the medical record. Maternal race, smoking history, maternal and paternal education (academic years achieved at enrollment), average annual household income based on residence postal zip code (http://www.marc.org), and infant feeding during the first four months (breastfeeding, formula-feeding, or both) were collected using a self-reported questionnaire as part of the parent study. 

Maternal blood samples were collected at enrollment in tubes containing EDTA to prevent coagulation. Plasma was separated at 4000× *g* at 10 °C and stored at −80 °C until analysis of 25(OH)D concentration by an enzyme-linked immunosorbent assay (ELISA) kit (Immundiagnostik AG, Bensheim, Germany; Alpco Diagnostics, Salem, NH, USA) in the Kansas Intellectual and Developmental Disabilities Research Center core laboratory. As we reported [17], the ELISA was performed according to manufacturer’s guidelines, which included a step to separate any bound 25(OH)D from the vitamin D binding protein. All samples were assayed in duplicate. The ELISA detected 25(OH)D in the range of 12 to 240 nmol/L with a 100% and 68% specificity toward 25(OH)D_3_ and 24(OHY)D_2_, respectively. Intra- and inter-assay coefficients of variation (CV) were both 7%. All samples obtained had a 25(OH)D concentration in the range of the assay. Red blood cell (RBC) phospholipid DHA was also measured at delivery, and the methods of analysis have been reported [18]. Data on frequency and type of infant infections at 6 and 12 months were extracted from medical records and coded into four infection categories: EENT (eye, ear, nose, and throat), respiratory, skin, and all others. These data were double-checked by a second laboratory member for accuracy.

### 2.3. Statistical Analysis

Subject characteristics were analyzed using descriptive statistics, and the normality of data distribution was tested using Kolmogorov–Smirnov test. Differences between AA and non-AA groups for continuous measures of plasma 25(OH)D concentrations were determined using Mann–Whitney *U*-test, and differences in sociodemographic characteristics and vitamin D status (deficient (<50 nmol/L), insufficient (50–75 nmol/L), and sufficient (≥75 nmol/L)) were determined using Chi-Squared test. Logistic regression modelling adjusting for maternal RBC-DHA concentration was used to compare the frequency of infants with at least one incidence of infection during the first 6 and 12 months of life in AA and non-AA groups. We used Spearman correlation to test the correlation between number of infections and maternal plasma 25(OH)D concentrations and between the number of infections and maternal RBC-DHA concentrations. We also used Spearman correlation to test the correlation between maternal education, household income, and maternal plasma 25(OH)D concentration. Univariate analysis of variance (ANOVA) was used to test differences in frequency of infections between infant feeding groups. We explored the determinants of having at least one incident infection in the first 6 months of life, including maternal education, income by zip code, and plasma 25(OH)D concentration as possible explanatory factors, and stratified by ethnicity. Statistical analyses were done using SAS, and a *p*-value < 0.05 was considered statistically significant. 

## 3. Results

### 3.1. Baseline Characteristics and Maternal Vitamin D Status

AA women were almost one week farther along in their pregnancy at the time enrollment blood was drawn for 25(OH)D compared to non-AA women (Supplementary Table S1). Forty three percent of both AA and non-AA women reported smoking at some time before pregnancy. Maternal education, paternal education, and estimated annual income by zip code were significantly lower in the AA group compared to the non-AA group (Supplementary Table S1). Maternal RBC-DHA was 6.18 ± 2.27% at birth, with significantly higher concentrations in the non-AA compared to the AA group (6.52 ± 2.43% vs. 5.42 ± 1.66%, *p* < 0.001). Of note, RBC-DHA among pregnant women who participated in the KUDOS trial was 4.7 ± 1.3% in the placebo group and 7.3 ± 2.2% in the supplemented group [18]. Significantly fewer infants in the AA group were exclusively breastfed during the first 4 months of life compared to the non-AA group (8.70% vs. 43.0% respectively, *p* < 0.001), with 34.8% of infants in the AA group being exclusively formula-fed compared to 17.9% in the non-AA group. The remaining 56.5% and 39.1% of infants in the AA group and non-AA group, respectively, were both breastfed and formula-fed during the first 4 months of life.

Consistent with national data [19,20], vitamin D deficiency (plasma 25(OH)D < 50 nmol/L) was common in our study population, but was observed to be particularly high among AA women. The distribution of plasma 25(OH) vitamin D concentration was skewed right in both AA and non-AA women, with a higher mean than median concentration. Approximately 95% of AA and 70% of non-AA women had either deficient or insufficient vitamin D status (<75 nmol/L) (Table 1). However, most AA women with low 25(OH)D were deficient, while approximately half of non-AA women with low 25 (OH)D were deficient and the other one half were insufficient. 

### 3.2. Frequency of Infant Infections during the First 6 and 12 Months of Life

Significantly more infants of AA women were diagnosed with at least one infection in the first 6 months of life compared to infants of non-AA mothers; however, no difference between the racial groups was found at 12 months (Table 2). Respiratory infections were the most prevalent infections in both groups, occurring at least once in ~42% of infants during the first 6 months of life. Skin infections in the first 6 months tended to be more common among infants of AA mothers than non-AA women (Table 2).

### 3.3. Relationship between Maternal 25(OH)D and Infant Infection

Because maternal RBC-DHA concentration and infant feeding type differed between AA and non-AA groups, we first explored whether these biological factors are associated with number of infant infections (i.e., potential confounders). No significant associations were found between RBC-DHA concentration and number of all infections (*rho* = 0.031, *p* = 0.645 at 6 months; *rho* = 0.01, *p* = 0.877 at 12 months). Additionally, no significant differences in number of infections were found across feeding groups at 6 and 12 months. The median number of infections was 1 at 6 months for infants in the three feeding groups (ranges of infection frequency: 0–6 in the exclusively breastfed group, 0–5 in the formula fed group, and 0–7 in the group with mixed feeding; *p =* 0.126). At 12 months, the median number of infections was 3 (range: 0–13) in the exclusively breastfed group, 2 (range: 0–10) in the formula-fed group, and 3 (range: 0–11) in the group with mixed feeding (*p* = 0.692).

Among all study participants (*n* = 220), as maternal plasma 25(OH)D concentration decreased, the total number of infections that an infant had during the first 6 months of life increased. This inverse association was statistically significant (*rho* = −0.125; *p* = 0.033). When stratified by type of infection among all participants, maternal 25(OH)D concentration was inversely associated with the number of EENT infections (*rho* = −0.116; *p* = 0.043) and skin infections (*rho* = −0.137; *p* = 0.021) infants had in the first 6 months. None of these associations reached statistical significance at 12 months. When stratified by race, the number of EENT and skin infections were significantly inversely associated with maternal 25(OH)D concentrations in the non-AA group at 6 months (*rho* = −0.158, *p* = 0.026; *rho* = −0.159, *p* = 0.025, respectively). We did not find any relationship between maternal 25(OH)D and infection in the offspring of AA women, but the range of 25(OH)D in AA women was very narrow.

We modelled the association between 25(OH)D and incidence of at least one episode of any infection, accounting for the effects of education and income (socioeconomic status, SES). In the group as a whole, only maternal education predicted higher 25(OH)D (*p* = 0.05). With stratified models (AA and non-AA), this association appeared only in non-AA women, but was not significant in either group. Unfortunately, education, income, and race were all highly related to maternal 25(OH)D (Supplementary Tables S1 and S2), making it impossible to determine if lower vitamin D or lower SES were driving the risk of infant infection.

## 4. Discussion

Programming of the immune system during fetal development can influence innate and adaptive immune responses later in life [21]. The role of vitamin D in immune system development and regulation has been an active area of research since the discovery of vitamin D receptors on monocytes, dendritic cells, macrophages, and activated T and B cells in the early 1980s [22,23]. Vitamin D deficiency during pregnancy may influence immune-mediated disease development in the offspring [24]. While the mechanisms of action remain to be elucidated, maternal vitamin D is proposed to “program” the developing immune system by: (a) altering the mother’s immune-competence and therefore immunity transfer (i.e., transfer of specific immunoglobulins) from mother to fetus via the placenta; (b) acting directly on the placenta, such as inducing innate antimicrobial responses and inhibiting TNF-α-induced inflammation in human trophoblasts; and (c) partly dictating the amount of vitamin D transferred to the fetus, whereby vitamin D regulates the development of innate and adaptive immune defenses [1,2,3].

In this study, we sought to determine if maternal 25(OH)D—an indicator of maternal vitamin D status—at midpregnancy was a predictor of infant infectious illness. While we found such a relationship before controlling for SES, vitamin D status cannot be unlinked from maternal income, education, and race due to the high collinearity among these variables. Maternal education may well be linked to efforts to consume more vitamin D during pregnancy, and could also be related to other maternal actions that reduce the likelihood of skin and EENT infections. Importantly, lower vitamin D status is a possible explanation for the higher incidence of infection, and unlike lower education and income, it may be a more easily modified. To our knowledge, this study is the first to suggest a relationship between maternal 25(OH)D concentration in pregnancy and infant infectious illness in a US cohort. Higher vitamin D status in pregnancy was linked to significantly fewer EENT and skin infections in the offspring early in infancy in the group of infants as a whole, as well as in infants of non-AA women. While AA women had poorer vitamin D status during pregnancy and their offspring had more infections than the offspring of non-AA women, we found no association between plasma 25(OH)D and infection or SES and infection in the AA group. Either these relationships do not exist, or they cannot be elucidated among women who are AA, due to the narrow range in plasma 25(OH)D concentration. The range of vitamin D concentration (5–130 nmol/L) in AA women was nearly half that seen in non-AA participants in this sample (14–226 nmol/L). Randomized trials to improve vitamin D status are needed to determine if improving vitamin D status can reduce infant infection. Indeed, it is well-established that maternal vitamin D supplementation at 4000 IU/day from 12–16 weeks gestation through delivery is effective at reducing vitamin D deficiency and insufficiency and is safe [25]. As well, benefits of supplementation at 4000 IU/day may extend beyond reducing the incidence of pre-eclampsia and maternal diabetes, and potentially include lasting benefits on the infant’s immune competence [26]. Several studies in other countries have evaluated respiratory infections in infancy in relation to maternal or newborn vitamin D status [6,7,8,9,10]. Gale et al. [6] found no significant association of 25(OH)D concentration with the development of at least one respiratory infection based on a parent report in a study from the UK; however, they did associate maternal 25(OH)D <30 nmol/L compared to 25(OH)D >75 nmol/L in late pregnancy with more eczema and childhood asthma. A large study (*n* = 922) from New Zealand found the odds of parent-reported respiratory infections in the first 3 months of life doubled in infants whose cord blood 25(OH)D was <25 nmol/L compared to those with cord blood 25(OH)D concentrations ≥75 nmol/L [7]. In a study from the Netherlands, Belderbos et al. [8] found a six-fold higher risk of respiratory syncytial viral infection during the first year of life in infants with cord blood 25(OH)D <50 nmol/L compared to >75 nmol/L. In studies from Egypt and Australia, infants hospitalized for acute lower respiratory tract infection during the first two years of life had cord blood 25(OH)D concentrations significantly lower than in infants not hospitalized for this condition [9,10]. More recently, the lowest risk for asthma and recurrent wheeze by 3 years of age was found in a group of children whose mothers were both supplemented with vitamin D (4400 IU/day) during pregnancy and had a plasma 25(OH)D concentration greater than 30 ng/mL before supplementation, compared to children whose mothers only received 400 IU/day of vitamin D and had initial 25(OH)D concentrations less than 20 ng/mL [16]. Interestingly, no racial differences in infant health outcomes were found due to vitamin D supplementation (*n* = 312 AA and *n* = 400 non-AA women) [16].

We found evidence that medically documented respiratory infections in the first 6 months were not related to maternal vitamin D status; this is consistent with Gale et al. [6], but contrasts with Camargo et al. [7]. We also did not find a racial difference in the incidence of respiratory infections. Respiratory infections in the first 6 months were common in both AA and non-AA infants, with 42% of infants in both groups having at least one respiratory infection. The high frequency of these infections and the lack of relationship to maternal 25(OH)D suggests innate immunity is not sufficiently protective. Most respiratory infections in infants and children are viral; respiratory viruses have many mechanisms to avoid recognition by the immune system [26], which could explain the apparent lack of protection of 25(OH)D against respiratory infections compared to skin and EENT infections.

Viral exposure, home environment, and/or the very young age of the infants could also be factors in our failure to find a protective effect of 25(OH)D status against respiratory infections [27]. We observed a range of maternal 25(OH)D concentrations in our population similar to those studied by Gale et al. [6] and Camargo et al. [7], so this does not appear to be a factor in the different findings of the three studies. It would have been of interest to compare the actual incidence of respiratory infections in the first 6 months in the three cohorts, had they included those results in their reports.

A limitation of this retrospective study is that we were not able to control for the infant’s vitamin D status at 6 and 12 months, which may mask the relationship between vitamin D exposure in utero and infections during months beyond the neonatal period. The infant’s vitamin D status may be affected by exposure to sunlight, oral vitamin D supplementation, dietary vitamin D composition (human milk, infant formula, complementary foods), and/or genetic nuances in vitamin D metabolism [20,28]. In addition to this missing data, our database did not include information on housing conditions or infants’ exposure to pollutants, allergens, tobacco, and lead. These environmental variables may be modifiers of disease risk when comparing health outcomes between racially diverse groups [29]. Maternal and paternal education as well as estimated annual household income were significantly lower in the AA group compared to the non-AA group. These differences were highly correlated with 25(OH)D in AA and non-AA women, making it impossible to disentangle SES from vitamin D status as a predictor of infectious illness. Our results should be interpreted within the scope of its secondary analysis design and associated limitations.

## 5. Conclusions

In summary, we found that compared to non-AA women, AA women had lower maternal 25(OH)D concentrations before mid-pregnancy and their infants had a higher incidence of EENT and skin infections in the first 6 months of life. Attempts to determine if the rate of infectious illness could be predicted by vitamin D status were complicated by the fact that vitamin D status, income, and education are so highly correlated. Nevertheless, we suggest that low vitamin D status in pregnancy remains a plausible biological mechanism for infectious illness in infancy in this cohort. Randomized trials to eliminate the high incidence of vitamin D deficiency and insufficiency in pregnancy in both AA and non-AA women could determine if infant infection is caused by lower vitamin D status in pregnancy.

## Figures and Tables

**Table 1:** Maternal vitamin D status during pregnancy.

	AA Group (*n* = 69)	Non-AA Group (*n* = 151)	*p*
Plasma 25(OH)D, nmol/L			
Mean ± SD	35.2 ± 22.7	63.2 ± 32.0	<0.001 ^2^
Median (IQR)	30.2 (17.9)	59.5 (35)	
5–95th percentile	7.38–70.4	23.7–117	
Vitamin D status ^1^, *n* (%)			
Deficient	58 (84.1)	56 (37.1)	<0.001 ^3^
Insufficient	8 (11.6)	55 (36.4)	
Sufficient	3 (4.30)	40 (26.5)	

AA: African American; 25(OH)D: 25-hydroxycholecalciferol; SD: standard deviation; IQR: interquartile range. ^1^ Based on Holick et al. (2011) [20]; ^2^ determined using Mann–Whitney *U*-test; ^3^ determined using Pearson Chi-Square test.

**Table 2:** Frequency of infants with at least one incidence of a medically-diagnosed infection during the first six and twelve months of life.

Infection	Infants of AA Group ^1^ *n* (%)	Infants of Non-AA Group *n* (%)	*p* ^2^
Any infection			
at 6 months	54 (78.3)	90 (59.6)	0.022
at 12 months	60 (90.0)	132 (87.4)	0.870
EENT infection			
at 6 months	26 (37.7)	41 (27.2)	0.226
at 12 months	36 (52.2)	92 (60.9)	0.277
Respiratory infection			
at 6 months	29 (42.0)	64 (42.4)	0.954
at 12 months	43 (62.3)	94 (62.3)	0.884
Skin infection			
at 6 months	22 (31.9)	24 (15.9)	0.055
at 12 months	32 (46.4)	55 (36.4)	0.342
Other infections			
at 6 months	10 (14.5)	16 (10.6)	0.946
at 12 months	18 (26.1)	36 (23.8)	0.997

AA: African American; EENT: eye, ear, nose and throat. ^1^ *n* = 69 infants in AA group and *n* = 151 infants in non-AA group. ^2^ *p*-value calculated from a logistic regression model adjusting for maternal RBC-DHA concentrations.

## Supplementary Materials

The following are available online at http://www.mdpi.com/2072-6643/10/2/111/s1. Table S1: Baseline parental characteristics; Table S2: Correlations between maternal education, annual household income by zipcode and maternal plasma 25(OH)D concentrations.
