# Supplementary material for: Maternal Vitamin D Status and Infant Infection
Source: Nutrients. 2018 Jan 23;10(2):111. doi: 10.3390/nu10020111 (PMC5852687; doi:10.3390/nu10020111)
Supplement: Supplementary file 1 [file nutrients-10-00111-s001.docx]

**Supplementary Table S1.** Baseline parental characteristics.

|  | **AA group** **(*n*** **=** **69)** | **Non-AA group** **(*n*** **=** **151)** | ***p*** |
| --- | --- | --- | --- |
| Gestational age, weeks | 15.2 ± 3.5 | 14.3 ± 3.5 | 0.06 ^1^ |
| Maternal education, years |  |  |  |
| Mean ± SD | 12.5 ± 1.7 | 15.0 ± 2.8 | <0.001 ^2^ |
| Median (IQR) | 12 (1) | 16 (5) |  |
| 5-95^th^ percentile | 10.4 - 15.6 | 11 - 20 |  |
| Paternal education, years |  |  |  |
| Mean ± SD | 12.4 ± 1.6 | 14.4 ± 2.9 | <0.001 ^2^ |
| Median (IQR) | 12 (0.625) | 14 (4) |  |
| 5-95^th^ percentile | 11 - 15 | 10 – 19.3 |  |
| Annual income, US$ |  |  |  |
| Mean ± SD | 35,430 ± 10,659 | 51,387 ± 18,121 | <0.001 ^2^ |
| Median (IQR) | 32,726 (15,068) | 49,065 (29,230) |  |
| 5-95^th^ percentile | 23,700 - 60,143 | 30,385 - 83,033 |  |

AA, African American; SD, standard deviation; IQR, interquartile range ^1^ determined using independent student *t*-test; ^2^ determined using Mann Whitney *U*-test.

**Supplementary Table S2.** Correlations between maternal education, annual household income by zipcode and maternal plasma 25(OH)D concentrations.

|  | Maternal Education, Years | Annual Income, US$ | Plasma 25(OH)D, nmol/L |
| --- | --- | --- | --- |
| Maternal Education, Years |  | *rho*= 0.562  *p* < 0.001 | rho=0.357  *p* < 0.001 |
| Annual Income, US$ | *rho*= 0.562  *p* < 0.001 |  | *rho*=0.402  *p* < 0.001 |
| Plasma 25(OH)D, nmol/L | *rho*=0.357  *p* < 0.001 | *rho*=0.402  *p* < 0.001 |  |

Correlations determined using Spearman correlation; *n* = 220.
